# Genetic Characterization of Hepatitis C Virus in Long-Term RNA Replication Using Li23 Cell Culture Systems

**DOI:** 10.1371/journal.pone.0091156

**Published:** 2014-03-13

**Authors:** Nobuyuki Kato, Hiroe Sejima, Youki Ueda, Kyoko Mori, Shinya Satoh, Hiromichi Dansako, Masanori Ikeda

**Affiliations:** Department of Tumor Virology, Okayama University Graduate School of Medicine, Dentistry, and Pharmaceutical Sciences, Shikata-cho, Okayama, Japan; Centro de Biología Molecular Severo Ochoa (CSIC-UAM), Spain

## Abstract

**Background:**

The most distinguishing genetic feature of hepatitis C virus (HCV) is its remarkable diversity and variation. To understand this feature, we previously performed genetic analysis of HCV in the long-term culture of human hepatoma HuH-7-derived HCV RNA-replicating cell lines. On the other hand, we newly established HCV RNA-replicating cell lines using human hepatoma Li23 cells, which were distinct from HuH-7 cells.

**Methodology/Principal Findings:**

Li23-derived HCV RNA-replicating cells were cultured for 4 years. We performed genetic analysis of HCVs recovered from these cells at 0, 2, and 4 years in culture. Most analysis was performed in two separate parts: one part covered from the 5′-terminus to NS2, which is mostly nonessential for RNA replication, and the other part covered from NS3 to NS5B, which is essential for RNA replication. Genetic mutations in both regions accumulated in a time-dependent manner, and the mutation rates in the 5′-terminus-NS2 and NS3-NS5B regions were 4.0–9.0×10^−3^ and 2.7–4.0×10^−3^ base substitutions/site/year, respectively. These results suggest that the variation in the NS3-NS5B regions is affected by the pressure of RNA replication. Several in-frame deletions (3–105 nucleotides) were detected in the structural regions of HCV RNAs obtained from 2-year or 4-year cultured cells. Phylogenetic tree analyses clearly showed that the genetic diversity of HCV was expanded in a time-dependent manner. The GC content of HCV RNA was significantly increased in a time-dependent manner, as previously observed in HuH-7-derived cell systems. This phenomenon was partially due to the alterations in codon usages for codon optimization in human cells. Furthermore, we demonstrated that these long-term cultured cells were useful as a source for the selection of HCV clones showing resistance to anti-HCV agents.

**Conclusions/Significance:**

Long-term cultured HCV RNA-replicating cells are useful for the analysis of evolutionary dynamics and variations of HCV and for drug-resistance analysis.

## Introduction

Hepatitis C virus (HCV) infection frequently causes chronic hepatitis, which progresses to liver cirrhosis and hepatocellular carcinoma. Such persistent infection has now become a serious health problem, with more than 170 million people worldwide infected with HCV [1]. HCV is an enveloped positive single-stranded RNA (9.6 kb) virus belonging to the *Flaviviridae* family, and the HCV genome encodes a large polyprotein precursor of approximately 3000 amino acid (aa) residues. This polyprotein is cleaved by a combination of host and viral proteases into at least 10 proteins in the following order: core, envelope 1 (E1), E2, p7, nonstructural protein 2 (NS2), NS3, NS4A, NS4B, NS5A, and NS5B [2], [3].

The initial development of a cell culture-based replicon system [4] and a genome-length HCV RNA-replicating system [5] using genotype 1b strains led to rapid progress in investigations into the mechanisms underlying HCV replication [6], [7]. HCV replicon RNA (approximately 8 kb) is a selectable, bicistronic HCV RNA with the first cistron, the neomycin phosphotransferase (Neo^R^) gene, being translated under control of the HCV internal ribosome entry site (IRES) and the second cistron, the NS3-NS5B regions, being translated under control of the encephalomyocarditis virus (EMCV) IRES. Genome-length HCV RNA (approximately 11 kb) possesses the Core-NS5B regions in substitution for the NS3-5B regions of the replicon in addition to the replicon structure. It was reported that infectious HCV particles are not produced in genome-length HCV RNA-replicating cell systems using genotype 1b strains [6], [8]. However, in 2005, an efficient virus production system using the JFH-1 strain of genotype 2a was developed using HuH-7-derived cells [9]. Since then, this infectious HCV system became a powerful tool to study the full viral life cycle [10].

The most distinguishing feature of the HCV RNA is its remarkable diversity and variation. To date, six major HCV genotypes, each having a large number of subtypes, have been found to show more than a 20% difference at the nucleotide level compared with any other genotypes [11], [12]. An approximately 5–8% difference at the nucleotide level has been observed within a single genotype [3]. Furthermore, an approximately 1% difference at the nucleotide level is also observed among HCV genomes in an individual [13]. Although genetic analyses of HCV using *in vivo* specimens have estimated that the genetic mutation rate of HCV is 1.4–1.9×10^−3^ base substitutions/site/year [14]–[16], the potential variability of HCV is not clear due to the selective pressure of immune system functions *in vivo* [17], [18].

To define the actual genetic mutation frequency of HCV, we previously performed genetic analysis of HCV [19], [20] using human hepatoma HuH-7 cell culture-based HCV replicon systems or genome-length HCV RNA-replication systems. In studies using the 1B-1 or O strain of genotype 1b, the accumulation of genetic mutations (mutation rate is 3.0–4.8×10^−3^ base substitutions/site/year), the enlargement of genetic diversity, and an increase in GC contents of HCV RNA were observed in a time-dependent manner during a 2-year cell culture [19], [20]. These results suggest that the long-term culture of HCV RNA-replicating cells is useful for understanding the evolutionary dynamics and variations of HCV. However, HuH-7-derived cells are the only cell culture system used thus far for robust HCV replication [6], [7]. Therefore, it remains unclear whether our results obtained from HuH-7-derived HCV RNA-replicating cell culture systems reflect the general features of HCV's genetic diversity and variation. On the other hand, in 2009 we established four new human hepatoma Li23 cell-derived genome-length HCV RNA (O strain of genotype 1b; GenBank accession no. AB191333)-replicating cell lines, OL (polyclonal; a mixture of approximately 200 clones), OL8 (monoclonal), OL11 (monoclonal), and OL14 (monoclonal) [21], and have been culturing them for more than 4 years. Since we demonstrated that the gene expression profile of Li23 cells was distinct from those in HuH-7 cells [22], and that anti-HCV targets in Li23-derived cells were distinct from those in HuH-7-derived cells [23]–[25], we expected to find distinct HCV variability and diversity from those observed previously in HuH-7-derived cells. To clarify this point, we carried out comprehensive genetic analysis of HCVs obtained from 0-year, 2-year, and 4-year cultures of OL, OL8, OL11, and OL14 cells, and compared them with the original ON/C-5B/QR,KE,SR RNA [21].

Here, we report the evolutionary HCV dynamics occurring in the long-term replication of genome-length HCV RNAs using Li23-derived cell culture systems.

## Materials and Methods

### Cell Cultures

The human hepatoma Li23 cell line, which was established and characterized in 2009, consists of human hepatoma cells from a Japanese male (age 56) [21]. The Li23 cells were cultured in modified medium for human immortalized hepatocytes, as described previously [21], [26]. Genome-length HCV RNA-replicating cells (Li23-derived OL, OL8, OL11, and OL14 cells) were cultured in the medium for the Li23 cells in the presence of 0.3 mg/ml of G418 (Geneticin, Invitrogen, Carlsbad, CA). These cells were passaged every 7 days for 4 years. HCV RNA-replicating cells possess the G418-resistant phenotype, because Neo^R^ as a selective marker was produced by the efficient replication of HCV RNA. Therefore, when HCV RNA is excluded from the cells or when its level decreases, the cells are killed in the presence of G418. In this study, OL, OL8, OL11, and OL14 cells were renamed OL(0Y), OL8(0Y), OL11(0Y), and OL14(0Y) cells, respectively, to specify the time at which the cells were established. These “0Y” cells of passage number 3 were used in this study. Two-year cultures of OL(0Y), OL8(0Y), OL11(0Y), and OL14(0Y) cells were designated OL(2Y), OL8(2Y), OL11(2Y), and OL14(2Y) cells, respectively. Four-year cultures of OL(0Y), OL8(0Y), OL11(0Y), and OL14(0Y) cells were designated OL(4Y), OL8(4Y), OL11(4Y), and OL14(4Y) cells, respectively.

### Quantification of HCV RNA

Quantitative reverse transcription-polymerase chain reaction (RT-PCR) analysis for HCV RNA was performed using a real-time LightCycler PCR (Roche Diagnostics, Basel, Switzerland) as described previously [21], [27]. Experiments were done in triplicate.

### Western Blot Analysis

The preparation of cell lysates, sodium dodecyl sulfate-polyacrylamide gel electrophoresis, and immunoblotting analysis with a PVDF membrane was performed as described previously [28]. The antibodies used to examine the expression levels of HCV proteins were those against NS4A (a generous gift from Dr. A. Takamizawa, Research Foundation for Microbial Diseases, Osaka University) and NS5B (a generous gift from Dr. M. Kohara, Tokyo Metropolitan Institute of Medical Science, Japan). Anti-β-actin antibody (AC-15; Sigma, St. Louis, MO) was also used to detect β-actin as an internal control. Immunocomplexes on the membranes were detected by enhanced chemiluminescence assay (Western Lightning Plus-ECL; Perkin-Elmer Life Sciences, Boston, MA).

### RT-PCR and Sequencing

To amplify genome-length HCV RNA, RT-PCR was performed separately in two fragments as described previously [21], [27]. Briefly, one fragment covered from the 5′-terminus to NS3, with a final product of approximately 5.1 kb, and the other fragment covered from NS2 to NS5B, with a final product of approximately 6.1 kb. These fragments overlapped at the NS2 and NS3 regions and were used for sequence analysis of the HCV open reading frame (ORF) after cloning into pBR322MC [28]. SuperScript II (Invitrogen, Carlsbad, CA) and KOD-plus DNA polymerase (Toyobo, Osaka, Japan) were used for RT and PCR, respectively. Plasmid inserts were sequenced in both the sense and antisense directions using Big Dye terminator cycle sequencing on an ABI PRISM 310 genetic analyzer (Applied Biosystems, Foster City, CA). The nucleotide sequences of each of 10 (OL cell series) or 3 (OL8, OL11, and OL14 cell series) independent clones obtained were determined.

### Molecular Evolutionary Analysis

Nucleotide and deduced aa sequences of the clones obtained by RT-PCRs were analyzed by neighbor-joining analysis using the program GENETYX-MAC (Software Development, Tokyo, Japan).

### Antiviral Assay

To monitor the anti-HCV activity of telaprevir, genome-length HCV RNA-replicating cells were plated onto 6-well plates (2×10^5^ cells for OL(0Y) cells or 8×10^4^ for OL(4Y), OL8(4Y), OL11(4Y), or OL14(4Y) cells per well). After 24 hrs in culture, the cells were treated with telaprevir (a generous gift from Dr. T. Furihata, Chiba University, Japan) at 0.2 μM or 0.4 μM for 3 days. After treatment, the cells were subjected to quantitative RT-PCR analysis for HCV RNA.

### Statistical Analysis

The significance of differences among groups was assessed using Student's *t*-test. *P*<0.05 was considered significant.

## Results

### Efficient replication of genome-length HCV RNA is maintained in long-term cell culture

**Figure 1:**
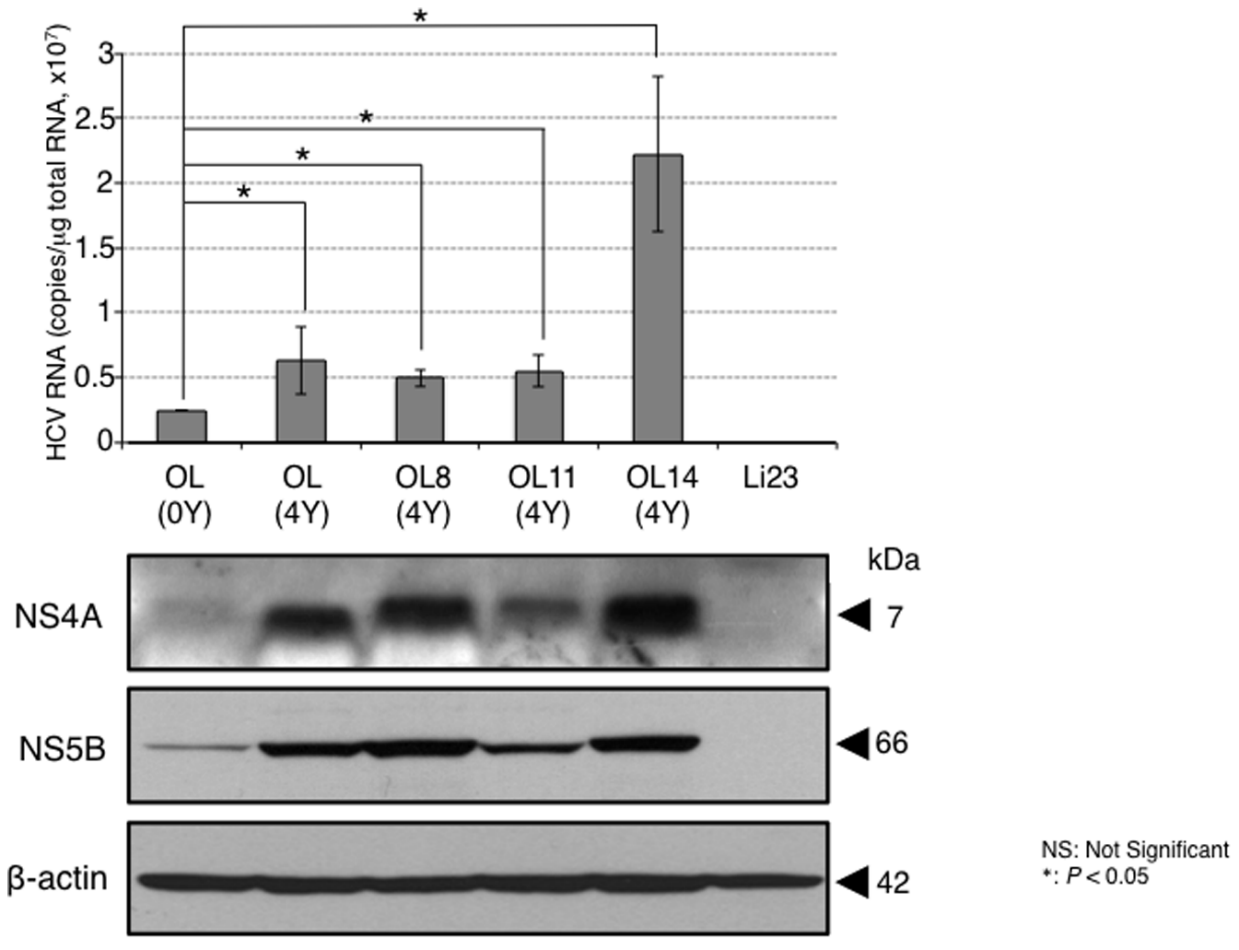
Characterization of genome-length HCV RNA-replicating cells after 4 years in culture. (A) Quantitative analysis of intracellular genome-length HCV RNA. The total RNAs from OL(4Y), OL8(4Y), OL11(4Y), and OL14(4Y) cells used were analyzed. The levels of intracellular genome-length HCV RNA were quantified by LightCycler PCR. OL(0Y) and Li23 cells were used as a positive and a negative control, respectively. (B) Western blot analysis. The cellular lysates from the cells used for RT-PCR analysis were also used for comparison. NS4A and NS5B were detected by Western blot analysis. β-actin was used as a control for the amount of protein loaded per lane.

To prepare the specimens for the genetic analysis of HCV, genome-length HCV RNA-replicating OL(0Y), OL8(0Y), OL11(0Y), and OL14(0Y) cells were cultured for 4 years. Since we previously demonstrated that the levels of HCV RNAs increased in all cases after 2 years of constitutive HCV RNA replication [26], in the present study we examined the levels of intracellular HCV RNAs after the cell culture of 4 years by quantitative RT-PCR. The results revealed that the levels of HCV RNAs in all cases were significantly higher than that of OL(0Y) cells (Fig. 1). Western blot analysis for HCV NS4A and NS5B also showed that the expression levels in all cases were higher than that of OL(0Y) cells. However, the present results were matched with previous findings regarding a 2 year-culture [26], revealing that the levels of HCV RNAs of OL8(4Y) and OL11(4Y) cells become lower than those of OL8(0Y) or (2Y) and OL11(0Y) or (2Y) cells, respectively. Unlike the results for the OL8 or OL11 series, the levels of HCV RNAs of OL(4Y) or OL14(4Y) cells were each maintained throughout cultures of 2 years and 4 years. Overall, we showed that the HCV RNA levels in all cases were more than 5×10^6^ copies/μg of total RNA, indicating that efficient HCV RNA replication occurred during those 4 years.

We next examined whether infectious HCV particles are produced from genome-length HCV RNA-replicating cells after 4 years of culture, although it has been reported that infectious particles were not produced in genome-length HCV RNA-replicating cell systems [6], [8]. To clarify this point, we performed infection experiments to HCV (JFH-1) susceptible HuH-7-derived RSc and Li23-derived ORL8 cells [21] using the supernatant of OL(0Y), OL(4Y), OL8(4Y), OL11(4Y), or OL14(4Y) cells as an inoculum. At 7 days and 8 days post-infection, we quantified the Core in the supernatants by enzyme-linked immunosorbent assay and HCV RNA in the cells by quantitative RT-PCR. The results (Fig. S1) showed that both Core and HCV RNA were not detected in our long-term cultured cells, suggesting that the cells produced no infectious virus particles over time.

### Genetic variations of genome-length HCV RNAs during long-term cell culture

**Figure 2:**
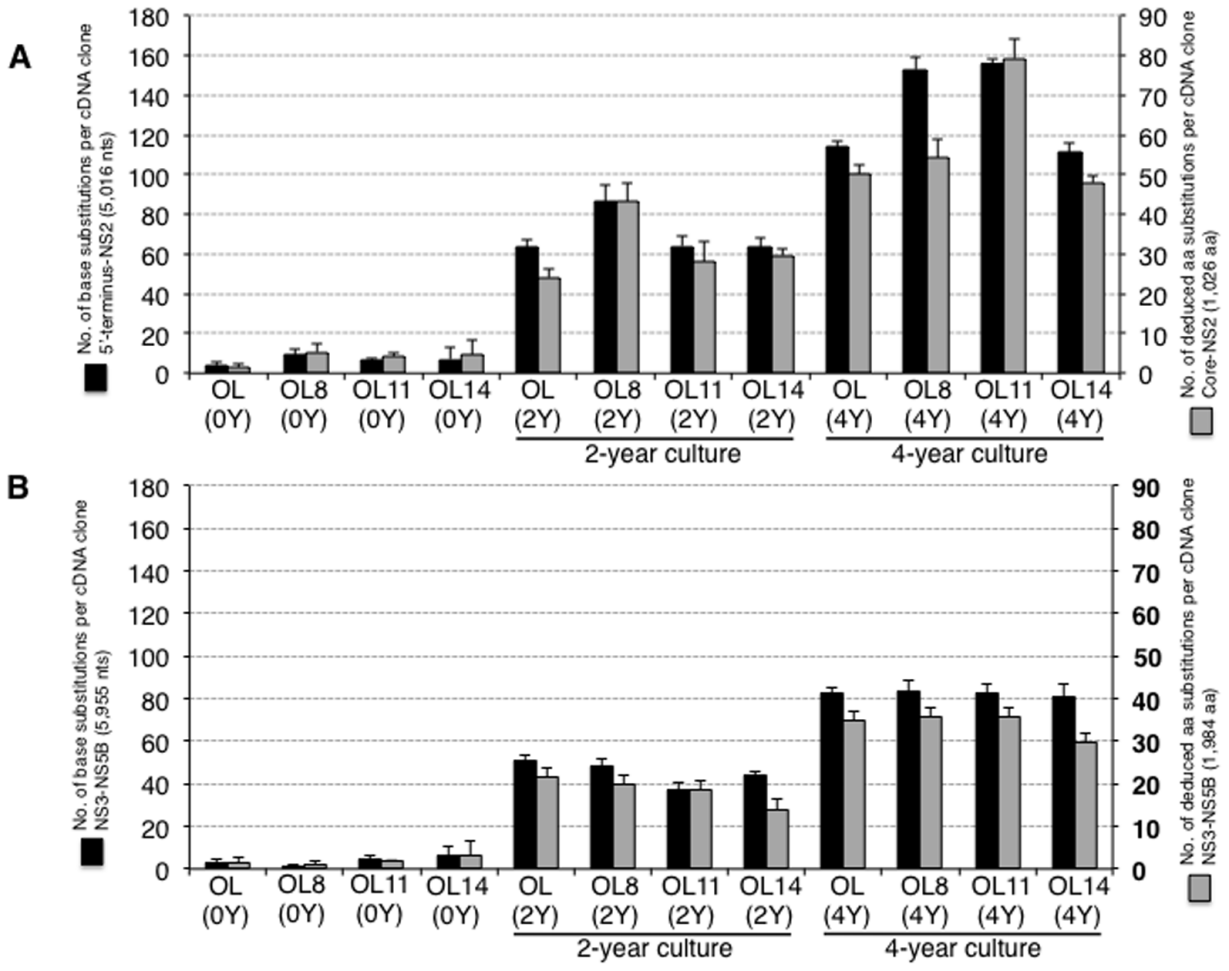
Genetic variations occurring in long-term replication of genome-length HCV RNAs. (A) Genetic variations in the 5′-terminus-NS2 regions. The left vertical line indicates the mean numbers of base substitutions detected per cDNA clone, by comparison with ON/C-5B/QR,KE,SR RNA [21]. The right vertical line indicates the mean numbers of aa substitutions in the Core-NS2 regions deduced per cDNA clone, by comparison with the original aa sequences deduced from ON/C-5B/QR, KE, SR RNA [21]. (B) Genetic variations in the NS3-NS5B regions. The mean numbers of base substitutions and aa substitutions are indicated as shown in (A).

**Figure 3:**
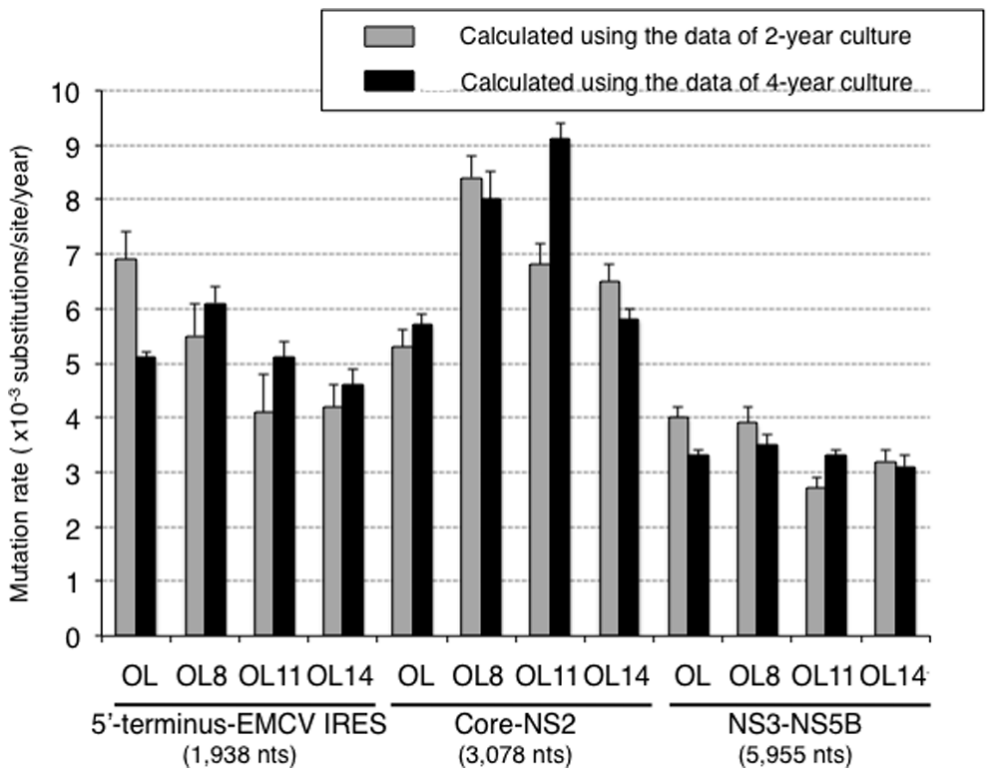
Mutation rates of genome-length HCV RNAs in long-term cell culture. The mutation rates of three regions (5′-terminus-EMCV-IRES, Core-NS2, and NS3-NS5B) of genome-length HCV RNAs (OL, OL8, OL11, and OL14) were calculated using the sequence data obtained from 2- or 4-year cell culture. The vertical line indicates the means of the mutation rates calculated using the nucleotide sequences of 10 clones (OL) or 3 clones (OL8, OL11, or OL14) of genome-length HCV RNAs, by comparison with the original sequence (ON/C-5B/QR,KE,SR RNA) [21].

To clarify the genetic variations of HCVs during the period of cell culture, we carried out sequence analysis of genome-length HCV RNAs obtained from OL(2Y), OL(4Y), OL8(2Y), OL8(4Y), OL11(2Y), OL11(4Y), OL14(2Y), and OL14(4Y) cells. The determined nucleotide sequences of genome-length HCV RNAs were compared with those of the original ON/C-5B/QR,KE,SR RNA [21] used for the establishment of the OL(0Y), OL8(0Y), OL11(0Y), and OL14(0Y) cell lines. To compare the nucleotide sequences, the data on genome-length HCV RNAs from OL(0Y), OL8(0Y), OL11(0Y), and OL14(0Y) cells were also used [21]. Most of the sequence analysis was performed in two separate parts: one part covers from the 5′-terminus to NS2, which is mostly nonessential for RNA replication, and the other part covers from NS3 to NS5B, which is essential for RNA replication. The results revealed that the numbers of base substitutions in both regions increased in a time-dependent manner (Fig. 2A and 2B). The numbers of deduced aa substitutions in HCV ORFs correlated well with the numbers of base substitutions of genome-length HCV RNAs (Fig. 2A and 2B). These base substitutions were considered mutations that occurred during the intracellular replication of genome-length HCV RNA. Based on the results after 2 or 4 years in culture, we calculated the apparent mutation rates of genome-length HCV RNAs in these cell lines. For this analysis, genome-length HCV RNA was divided into three parts: the 5′-terminus-EMCV IRES regions (partly essential for RNA replication), the Core-NS2 regions (nonessential for RNA replication), and the NS3-NS5B regions (essential for RNA replication). The results revealed that the mutation rates (base substitutions/site/year) in the three distinct regions calculated from the data of the 2-year culture were about the same as the mutation rates calculated from the data of the 4-year culture (Fig. 3). These results suggest that genetic variations of HCV have occurred at the same speed for four years in Li23-derived genome-length HCV RNA replicating cells. Furthermore, we noticed that the mutation rates in the NS3-NS5B regions (2.7–4.0×10^−3^) were lower than those in the 5′-terminus-EMCV IRES regions (4.1–6.9×10^−3^) and the Core-NS2 regions (5.3–9.1×10^−3^) (Fig. 3). Moreover, we examined the numbers of synonymous (dS) and nonsynonymous (dN) mutations with transition (Ts) or transversion (Tv) in two divided regions (Core-NS2 and NS3-NS5B). The results are summarized in Table 1. The dN/dS ratio in the Core-NS2 and NS3-NS5B regions were 1.55 to 3.00 and 0.45 to 1.06, respectively. These values imply the positive selection in Core-NS2 regions and the purifying (stabilizing) selection in NS3-NS5B regions except OL11(2Y) and OL8(4Y) cells. Since the dN/dS ratios in NS3-NS5B regions of OL11(2Y) and OL8(4Y) cells were 1.06 and 1.03, respectively, we can estimate that neutral selection acted in these cells. In addition, the Ts/Tv ratios in the Core-NS2 and NS3-5B regions were 3.50 to 7.21 and 3.58 to 10.08, respectively. These results showed a tendency similar to that found in a previous study [20] using HuH-7-derived genome-length HCV RNA-replicating cells, suggesting that the NS3-NS5B regions, which are essential for RNA replication, are evolutionally limited. Together these results indicate that HCV can mutate at the same level in both HuH-7-derived cells and Li23-derived cells.

**Table 1:** Base substitutions occurring in genome-length HCV RNAs during long-term cell culture.

Full-length HCV RNA series	Ts				Tv				dN/dS		Ts/Tv	
	dN		dS		dN		dS					
	C-NS2	NS3-5B	C-NS2	NS3-5B	C-NS2	NS3-5B	C-NS2	NS3-5B	C-NS2	NS3-5B	C-NS2	NS3-5B
OL(2Y)	21.2±1.4	11.5±1.4	9.1±1.5	29.3±2.0	3.1±1.4	9.0±1.5	1.1±0.3	0.5±0.5	2.38	0.69	7.21	4.29
OL8(2Y)	34.3±4.9	14.3±1.2	12.3±2.1	23.7±.25	7.7±0.6	5.7±1.2	1.7±0.6	4.3±0.6	3.00	0.71	5.00	3.80
OL11(2Y)	23.3±4.0	13.0±1.0	17.0±3.6	16.0±4.0	6.0±3.6	6.0±1.7	0.7±0.6	2.0±0	1.66	1.06	6.05	3.63
OL14(2Y)	18.7±1.5	11.0±1.0	16.3±2.1	29.3±4.7	8.7±0.6	2.7±2.9	1.3±0.6	1.3±1.5	1.55	0.45	3.50	10.08
OL(4Y)	47.4±3.2	22.1±1.7	16.4±2.0	45.1±2.5	5.1±0.9	13.1±1.2	4.0±0.5	2.3±0.5	2.57	0.74	7.01	4.36
OL8(4Y)	56.7±4.2	35.7±1.2	29.7±2.5	38.3±2.3	14.3±0.6	12.3±0.6	1.3±0.6	8.3±0.6	2.29	1.03	5.51	3.58
OL11(4Y)	66.7±4.9	26.3±0.6	30.0±5.6	42.0±3.6	16.3±2.9	6.7±1.5	4.3±0.6	6.7±3.2	2.42	0.68	4.68	5.13
OL14(4Y)	34.3±1.5	23.7±1.2	27.3±3.5	47.3±2.9	10.3±1.2	5.0±0	1.3±0.6	3.7±1.5	1.56	0.56	5.29	8.19

Base substitutions were counted by comparison with the sequence of genome-length HCV RNA (ON/C-5B/QR,KE,SE [20]).

Average numbers of base substitutions per cDNA clone are shown.

Ts: Transition; Tv: Transversion; dN: Nonsynonymous; dS: Synonymous.

### Characterization of aa substitutions in HCV ORFs during long-term cell culture

We next characterized aa substitutions in HCV ORFs that occurred during 4 years in culture of OL(0Y), OL8(0Y), OL11(0Y), and OL14(0Y) cells. The conserved aa substitutions (mutated in all 10 clones sequenced in the cases of OL(2Y) or OL(4Y) cells and mutated in all 3 clones sequenced in the cases of OL8(2Y), OL8(4Y), OL11(2Y), OL11(4Y), OL14(2Y), or OL14(4Y) cells) are summarized in Table 2 (Core-p7 regions) and Table 3 (NS2-NS5B regions). Among the many aa substitutions, only 19 were the same as those detected in the 2-year culture of one of five kinds of HuH-7-derived genome-length HCV RNA (O strain of genotype 1b)-replicating cell lines [20] (Tables 2 and 3). In addition, 17 aa were substituted to the type of JFH-1 strain (genotype 2a: accession number AB237837) (Tables 2 and 3). We noticed that 12 aa substitutions were commonly detected in at least two different cell lines (Tables 2 and 3). The remaining 338 conserved aa substitutions were independently caused in each of the Li23-derived genome-length HCV RNA-replicating cell lines (Tables 2 and 3). However, from these results, we cannot conclude it whether genetic variations of HCV occur in a cell-line-specific manner or in a random manner.

**Table 2:** Conservative aa substitutions occurring during long-term replication of genome-length HCV RNAs (I).

	OL		OL8		OL11		OL14	
Region								
Core	V46A	**T52A**	K10R^a^^,^ b	T11S	(I30T)	**S53P**	K10R^a^^,^ b	**K12N** b
(1∼191)	L133F^b^	**G146R**	**Q20R**	V31A	T125S	**L133S**	**K23M**	E54G
	**N163D**	**L185S**	W76R	E89V^b^	M134T	**L139P** b	**S56P**	I65V
			L91P	N118I	A150T	N163T^a^^,^ b	A180V	
			E159V	N163T^a^^,^ b				
			P170A					
E1	**Y201H**	Y214C	C207Y	**V230A**	D206G	V240L	V203I	**C226R** b
(192∼383)	D218T	**L246P**	C281Y	**V284A**	A241T	S251G^a^	S251G^a^	**Y276H**
	**F271S**	I287N	**L286P**	F293L	S283P	**V284G**	L308S	**A343V** c
	Y298H^c^	C306S	S294L	**V313A** b	C304R	**M318V** a	**A380S**	
	W320R	**L332P**	(M318V)^a^	M323L	V365A	L377F		
	**L359F**		**T329A**	**L338F**				
			Q342R	V344G				
			**A351P**	**S363P**				
			**W368R**	F378L				
E2	R386C	I414T	**R386H**	N395D	S408P	R424G	I411V	**I414Δ**
(384∼746)	S450P	M456T	K410E	**N417D**	**L427P**	(G436E)	**S419R**	I422T
	**E464A** c	N532G	**N428D**	(I462V)	F447L	(S449P)	R483G	D520G
	**N556S**	K596E	I462A	**D481E**	S449L	**F465L**	K562E	T563M
	R614G	**M631T**	**Y507H** b	**G523S**	(Q467H)	V514G	C564W	**T680S** c
	E650G	L692P	L537P^b^	**N548S** c	E533G	C569R	**D698G**	V699A
	V710A	L721P^a^	**T561S**	E591G	N577T^c^	(L603M)	Δ725–746	
			S668P	I674T^b^	V609I	D610G		
			**Δ686–702**	**V709A**	Y611C	W616R		
			W736R		N623S	S663G		
					F679L	V710I		
					**V712A**	L721P^a^		
					**V731A**			
p7	E749K	**G764S**	**S767P**	(L797I)	N750D	L766F	(L748P)	Δ747–759
(747∼809)	**L769P**				L799P		**F771L**	**I778V**

a Conservative aa substitutions detected in at least two of four cell line series.

b Conservative aa substitutions detected in HuH-7-derived cell line series (O, OA, OB, OD, or OE) used in the previous study [20].

c Conservative aa substitutions that became the same aa as the JFH-1 strain.

Conservative aa substitutions detected after 2-year and 4-year cultures are shown by bold letters.

Conservative aa substitutions detected only after 2-year culture are shown within parentheses.

**Table 3:** Conservative aa substitutions occurring during long-term replication of genome-length HCV RNAs (II).

	OL		OL8		OL11		OL14	
Region								
NS2	Y835H	F886L^b^	**M814I**	**I824V**	W845R	**V853A**	F823S	**W844R**
(810∼1026)	**L892S**		L849F	**R852G**	D871G	**T877A**	**Q847H**	**Y848Δ**
			**A855T**	**Q903R** a ^,^ c	I885T	(P898L)	Q903R^a^^,^ c	I983T
			K927R	E1019G	Q903R^a^^,^ c	V913A		
					L924S			
NS3	V1081A	**E1202A**	**P1122S**	V1415I	**S1173L**	M1205V	M1268V	P1290H
(1027∼1657)					**T1280A**	(I1412V)	D1581G	R1596K
					**F1501Y** c	Q1606R	A1647T^c^	
					F1644L			
NS4A	Q1703R							
(1658∼1711)								
NS4B	**S1827T** c	**V1880A** b			I1769V	**Q1804R**	A1743V	S1827A
(1712∼1972)	**P1908L**	L1956M			Q1955R		V1906A	
NS5A	**L2003F**	H2057R	**R1978K**	D1979E	K2050R	**F2099Y** c	L2125V^c^	**D2220G** a
(1973∼2419)	S2246P	I2252S	K1998R	S2079Y	**T2217I**	I2274V	F2281L^a^^,^ c	**D2292E** a ^,^ b
	T2278A	**F2281L** a ^,^ c	K2212R^c^	**D2220G** a	K2277R	S2283P^a^	F2352L	S2355T
	S2283P^a^	**D2292E** a ^,^ b	**E2263G**	**E2265V**	K2320R^a^	**T2336S** c	S2373P	D2374N
	K2320R^a^	S2338P	V2270A	K2280D	T2351A	**F2352S**	A2382V	G2396R
	**S2355P**	P2369H	**Y2293H**	D2305N	**W2405R** a ^,^ b		**S2401N**	W2405R^a^^,^ b
	**S2384P**	**M2388T**	S2342P^b^	L2347R			**C2418R** b	
	**G2403R**	**S2409R**	F2352V	T2364A				
			D2377G^b^	**S2380T**				
			D2381G	S2387P^b^				
			**W2405R** a ^,^ b	S2406A				
			E2410K					
NS5B	K2470R	**D2771N** c	S2975G^c^	**I3004V**	K2493R	T2549A	**A2444T**	**H2539R**
(2420∼3010)	L2853I	Q2933R			K2689R	Q2728R	V2918I	
	**V3000A**							

a Conservative aa substitutions detected in at least two of four cell line series.

b Conservative aa substitutions detected in HuH-7-derived cell line series (O, OA, OB, OD, or OE) used in the previous study [20].

c Conservative aa substitutions that became the same aa as JFH-1strain.

Conservative aa substitutions detected after 2-year and 4-year cultures are shown by bold letters.

Conservative aa substitutions detected only after 2-year culture are shown in parentheses.

### Genetic deletions were characterized in the first half of genome-length HCV RNAs during long-term cell culture

**Figure 4:**
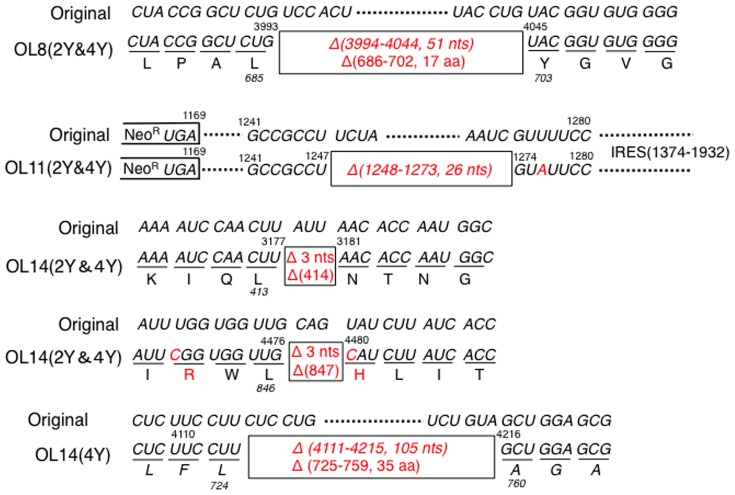
Genetic deletions occurred in the first half of genome-length HCV RNAs during the long-term cell culture. The conservative deleted portions in the genome-length HCV RNAs derived from OL8(2Y), OL8(4Y), OL11(2Y), OL11(4Y), OL14(2Y), or OL14(4Y) cells were shown by boxes. The original sequence was from ON/C-5B/QR,KE,SR RNA [21].

Recently, Pacini et al. demonstrated that naturally occurring HCV subgenomic RNAs, mostly lacking the E1 or E2 region, were capable of autonomous replication and could be packaged and secreted in viral particles [29]. In the present cell-based study also, we detected several conserved deletions within genome-length HCV RNAs, although a previous study using HuH-7-derived cell lines did not reveal any conserved deletions [20]. As shown in Figure 4, all deletions were located in the first half of genome-length HCV RNA. In OL8(2Y) and OL8(4Y) cells, a conserved 51 nucleotides (nts) deletion in frame was detected, resulting in a 17 aa deletion (aa 686–702 in the E2). In OL14(2Y) and OL14(4Y) cells also, two kinds of conserved 3 nts deletion in frame were detected, resulting in a 1 aa deletion in each (aa 414 in the E2 and aa 847 in the NS2). Furthermore, a conserved 105 nts deletion in frame was observed in OL14(4Y) cells, resulting in a 35 aa deletion (aa 725–746 in the E2 and aa 747–759 in the NS2). In addition, 26 nts (nt 1248–1273) located between the *Neo^R^* gene and IRES was conservatively deleted in OL11(2Y) and OL11(4Y) cells. These results suggest that nonessential regions for RNA replication are deleted during long-term culture of Li23-derived cells. However, such deletion was not caused in the OL cell series.

### Genetic diversity of genome-length HCV RNA arising during long-term cell culture

**Figure 5:**
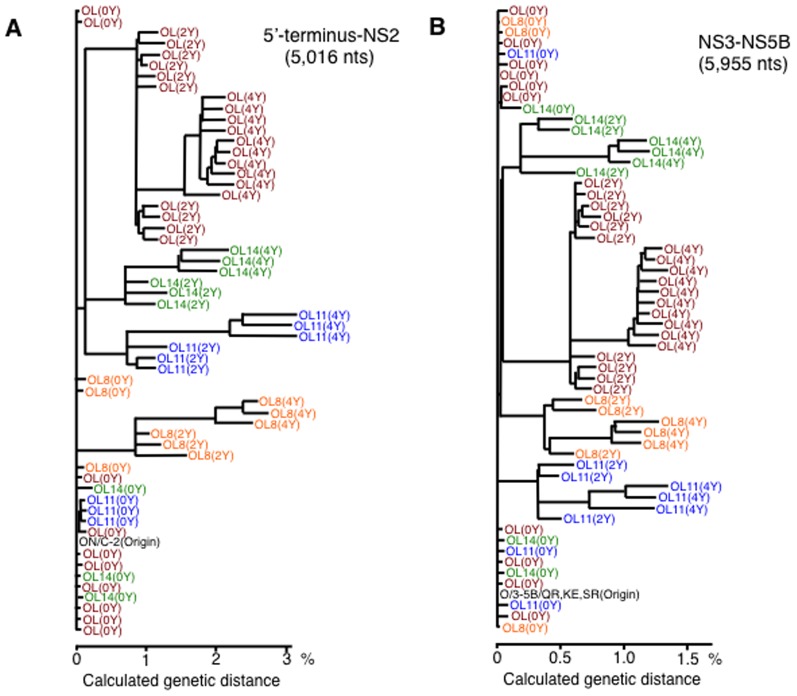
Phylogenetic trees of genome-length HCV RNA populations obtained in long-term cell culture. The phylogenetic trees are depicted on the basis of nucleotide sequences of all cDNA clones obtained by 0-year, 2-year, and 4-year cultures of OL, OL8, OL11, and OL14 cells. (A) The 5′-terminus-NS2 regions of genome-length HCV RNA. ON/C-2 indicates the original sequences of the 5′-terminus-NS2 regions of ON/C-5B/QR,KE,SR RNA [21]. (B) The NS3-NS5B regions of genome-length HCV RNA. O/3-5B/QR,KE,SR indicates the original sequences of the NS3-NS5B regions of ON/C-5B/QR,KE,SR RNA [21].

Based on the sequence data of all clones obtained after 0-year, 2-year, and 4-year culture, we examined the genetic diversities of genome-length HCV RNAs by the construction of phylogenetic trees. The results revealed that the genetic diversities of genome-length HCV RNAs were clearly expanded at both the nucleotide (Fig. 5) and aa (Fig. S2) sequence levels in the 5′-terminus-NS2 regions and the NS3-NS5B regions, and that the 10 clones derived from OL cell series and 3 clones derived from each other cell series were clustered and located at similar genetic distances from the origin (ON/C-2 or O/3-5B/QR,KE,SR for the nucleotide sequence level and O/C-2 or O/3-5B/QR,KE,SR for the aa sequence level [21]) (Fig. 5 and Fig. S2).

We next compared the nucleotide sequences among 10 independent OL(4Y) clones obtained after 4-year cell culture. In the 5′-terminus-NS2 regions and the NS3-NS5B regions derived from OL(4Y) cells, 0.38–1.28% and 0.22–0.56% differences in nucleotide sequences were observed, respectively. These results suggest that the quasispecies nature of genome-length HCV RNA was acquired steadily over long-term intracellular RNA replication.

### Classification of mutations occurred in genome-length HCV RNAs during long-term cell culture

**Figure 6:**
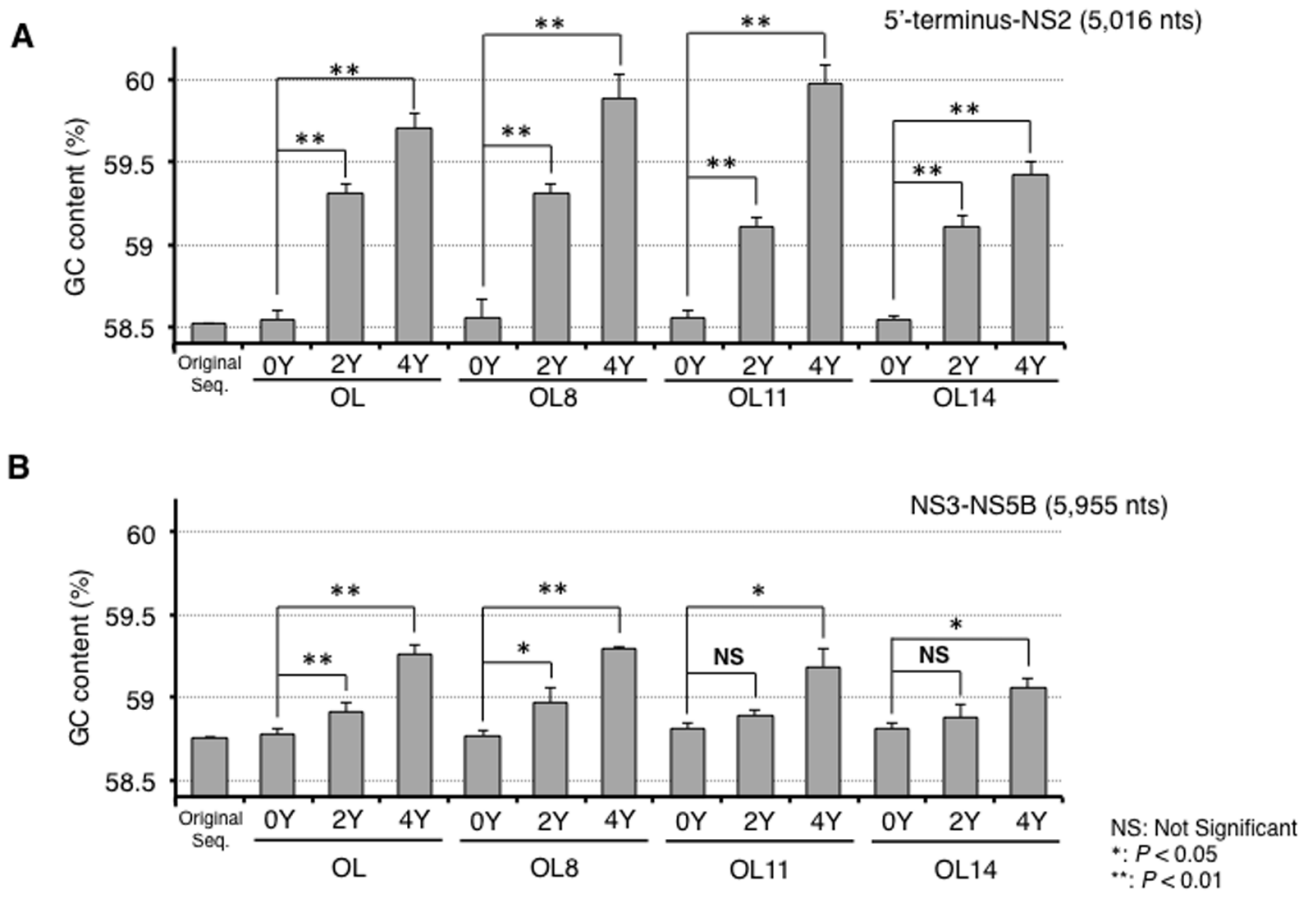
Increased GC content of genome-length HCV RNAs occurring in long-term RNA replication. The GC content of cDNA clones obtained by 0-year, 2-year, and 4-year culture of OL, OL8, OL11, and OL14 cells was calculated. The values indicate the means of 10 clones (OL) or 3 clones (OL8, OL11, or OL14). (A) The 5′-terminus-NS2 regions. (B) The NS3-NS5B regions.

We next examined the mutation patterns occurring in genome-length HCV RNAs. The results revealed that U to C and A to G transition mutations were the most and second-most frequent mutations in total, although three cases (OL8(2Y), OL8(4Y), and OL14(4Y)) showed the opposite result (Table 4). High frequencies of U to C and A to G mutations were also observed in a previous study using HuH-7-derived HCV replicon- or genome-length HCV RNA-replicating cell lines [19], [20]. The rarest mutation was C to G transversion in 2-year and 4-year cultures (Table 4). This result was the same as in a previous report using HuH-7-derived cell systems [20]. Since the frequency of U to C and A to G mutations was two or three times higher than that of C to U and G to A mutations, the GC content of HCV RNA increased significantly in a time-dependent manner in both the 5′-terminus-NS2 regions (Fig. 6A) and the NS3-NS5B regions (Fig. 6B). The increase in GC content of HCV RNA was observed in all Li23-derived cells after 2-year or 4-year culture. In the 5′-terminus-NS2 regions of HCV RNA, a remarkable (more than 1%) increase in GC content was found after the 4-year culture of all the cells except OL14(0Y) cells (Fig. 6A).

**Table 4:** Base substitution patterns occurred in genome-length HCV RNAs during the long-term cell culture.

	Average numbers of base substitutions per cDNA clone										
Base									Sum		HuH-7-derived
substitution	OL	OL	OL8	OL8	OL11	OL11	OL14	OL14	OL∼OL14	OL∼OL14	O, OA, OB, OD&OE
pattern	(2Y)	(4Y)	(2Y)	(4Y)	(2Y)	(4Y)	(2Y)	(4Y)	(2Y)	(4Y)	(2Y)*
Transition											
U →C	46.0	79.9	38.7	69.3	31.0	74.7	32.7	51.0	37.1±6.8	68.7±12.6	32.1±3.5
A →G	25.0	39.4	39.3	77.0	26.0	71.3	29.3	57.7	29.9±6.5	61.4±16.7	30.5±6.2
C →U	13.3	22.7	14.7	27.0	15.3	32.7	16.3	29.7	14.9±1.3	28.0±4.2	11.3±2.2
G →A	8.7	15.5	10.7	20.0	10.3	19.0	11.7	24.3	10.4±1.3	19.7±3.6	10.5±4.0
Transversion											
C →A	6.1	9.1	9.0	9.7	1.3	6.3	4.0	3.3	5.1±3.3	7.1±2.9	1.7±1.1
U →G	2.2	6.5	1.0	6.0	2.7	7.0	1.0	6.7	1.7±0.9	6.6±0.4	2.5±1.3
A →U	1.4	1.8	4.7	13.0	2.3	8.0	2.7	2.7	2.8±1.4	6.4±5.2	2.2±1.4
U →A	1.8	3.5	3.3	4.3	5.7	10.0	1.7	5.7	3.1±1.9	5.9±2.9	2.8±1.3
A →C	3.9	5.7	3.0	3.7	1.0	4.7	3.0	4.3	2.7±1.2	4.6±0.8	3.9±0.8
G →U	1.2	2.2	1.3	2.3	1.3	4.3	3.3	3.3	1.8±1.0	3.0±1.0	1.9±0.6
G →C	3.3	4.1	1.0	1.7	1.3	2.3	1.0	1.0	1.7±1.1	2.3±1.3	2.4±1.6
C →G	0.2	3.4	1.0	1.3	1.0	0.0	0.7	2.0	0.7±0.4	1.7±1.4	1.5±1.3

Base substitutions were counted by the comparison with the sequence of genome-length HCV RNA (ON/C-5B/QR,KE,SR [20]).

*Data from the previous study [20].

The time-dependent increase in the GC content of the HCV RNA may gradually change to an energetically stable form during RNA replication. We assumed that the increase in GC content is due to an increase in G- and C-ending codons, except for AGG and UUG codons, for efficient expression in human cells, so-called codon optimization [30], and we examined this possibility. The results in the NS3-NS5B regions revealed the time-dependent increase of G- and C-ending codons, except for AGG and UUG codons, in all four cell series (Table 5). However, this phenomenon was not remarkable in the Core-NS2 regions (Table 5). These results suggest that codon usage in the NS3-NS5B regions adapts to efficient translation in the human cells in a time-dependent manner. Further long-term cell cultures will clarify this point.

**Table 5:** Contribution degrees of the G- and C-ending codons except AGG and UUG codons in the GC content increase during 2-year or 4-year cell cultures.

C-NS2				
	OL	OL8	OL11	OL14
2Y culture	9.3*/24.0** (39%)	7.3/27.7 (26%)	4.3/20.6 (21%)	3.0/17.4 (17%)
4Y culture	9.8/38.1 (26%)	6.7/49.8 (13%)	17.7/54.7 (32%)	5.0/24.3 (21%)

NS3-5B				
	OL	OL8	OL11	OL14
2Y culture	2.1/9.0 (23%)	4.0/12.7 (31%)	0/7.9 (0%)	3.3/6.7 (49%)
4Y culture	12.5/29.9 (42%)	13.7/32.0 (43%)	6.7/25.8 (24%)	16.0/18.0 (89%)

*The increased numbers of G- and C-ending codons except AGG and UUG codons per cDNA clone.

**The increased numbers of G and C per cDNA clone.

### Usefulness of long-term cultured genome-length HCV RNA-replicating cells as a source of resistant HCV for anti-HCV agents

**Figure 7:**
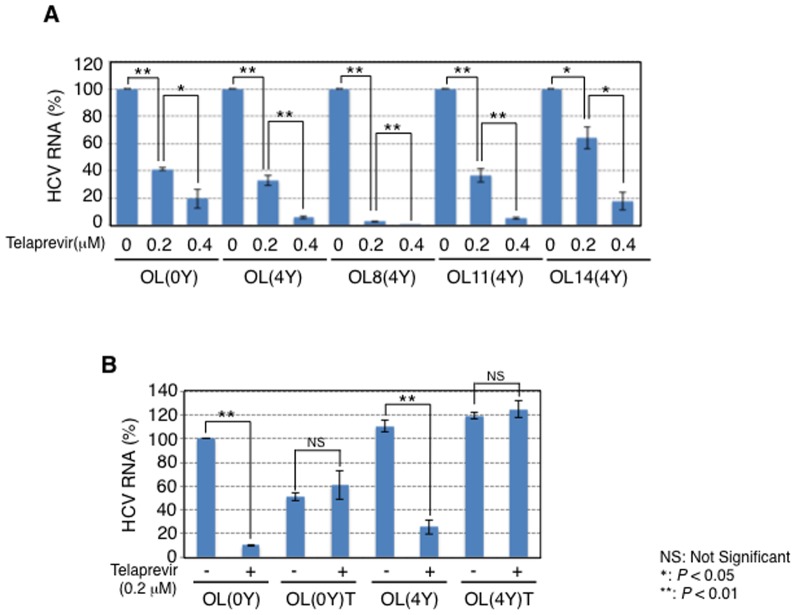
Sensitivity to telaprevir of the 4-year cultured genome-length HCV RNA-replicating cells. (A) Telaprevir sensitivities on genome-length HCV RNA replication in OL(4Y), OL8(4Y), OL11(4Y), and OL14(4Y) cells. OL(0Y) cells were used as a control. The cells were treated with telaprevir for 72 h, and then the levels of intracellular genome-length HCV RNA were quantified by LightCycler PCR. (B) Telaprevir-treated OL(0Y) and OL(4Y) cells (designated as OL(0Y)T and OL(4Y)T, respectively) became telaprevir-resistant easily. Telaprevir treatment and quantitative RT-PCR were preformed as shown in (A).

As described above, we demonstrated that genetic mutations and the diversity of HCV RNA expanded during long-term culture of genome-length HCV RNA-replicating cells. From these results, we assumed that these HCV populations that mimic the state of long-term persistent infection become the source of resistant HCV for anti-HCV agents. To clarify this point, we examined the effect of telaprevir, an inhibitor of HCV NS3-4A protease, which is the first directly acting antiviral reagent to be used for the treatment of HCV genotype 1, using 4-year cultured cell lines [31]. To know the effective concentration area, we first evaluated the anti-HCV activity of telaprevir using our previously developed HCV reporter assay systems (HuH-7-derived OR6 [27] and Li23-derived ORL8 [21]). The results revealed that 50% effective concentration (EC_50_) values were 0.17 μM and 0.14 μM in the OR6 and ORL8 assay systems, respectively, indicating that telaprevir exhibited strong anti-HCV activities in our HCV cell culture systems (data not shown). In reference to these EC_50_ values, we next examined the anti-HCV activity of telaprevir at 0.2 and 0.4 μM for 3 days on OL(4Y), OL8(4Y), OL11(4Y), and OL14(4Y) cells. OL(0Y) cells were also used as a control. Telaprevir at 0.2 and 0.4 μM inhibited approximately 60% and 80%, respectively, of HCV RNA replication on OL(0Y) cells, as expected from the results of the reporter assay, and that the anti-HCV activities of telaprevir on OL(4Y), OL11(4Y), and OL14(4Y) cells were similar to that on OL(0Y) cells (Fig. 7A). Unexpectedly, however, HCV RNA replication on OL8(4Y) cells was highly sensitive to telaprevir. Approximately 97% of HCV RNA replication was inhibited by 0.2 μM of telaprevir (Fig. 7A). These results suggest that HCV mutations that occur during long-term cell culture do not control the anti-HCV activity of telaprevir. Next we examined the possibility that long-term cultured cells can become the source of telaprevir-resistant HCV. First, OL(0Y) and OL(4Y) cells were treated with or without 0.4 μM of telaprevir (3 times at 6-day intervals) and 0.8 μM of telaprevir (3 times at 6-day intervals) in the presence of G418. The growth of the cells treated with telaprevir first slowed but then recovered. In this stage, we checked the anti-HCV activity of telaprevir at 0.2 μM for 3 days on telaprevir-treated OL(0Y) and OL(4Y) cells (designated OL(0Y)T and OL(4Y)T cells, respectively) with untreated OL(0Y) and OL(4Y) cells. The results clearly indicated that OL(0Y)T and OL(4Y)T cells completely converted telaprevir-sensitive phenotypes into telaprevir-resistant phenotypes (Fig. 7B). It is noteworthy that telaprevir-resistant OL(4Y)T cells were provided without a decrease in the level of HCV RNA replication. These results suggest that long-term cultured OL(4Y) cells may easily convert the phenotypes against anti-HCV drugs such as telaprevir.

## Discussion

In the present study, using Li23-derived cells unlike HuH-7, we characterized the genetic evolution and dynamics of HCV in the long-term culture of four kinds of genome-length HCV RNA-replicating cells, and demonstrated that genetic mutations of HCV accumulated and the genetic diversity of HCV expanded in a time-dependent manner. The GC content of HCV RNA was also significantly increased in a time-dependent manner. These phenomena, including the increased mutation rates, were consistent with those observed in the previous study using HuH-7-derived cell culture systems [19], [20]. However, we detected several in-frame deletions in the structural regions, suggesting that the environment maintaining RNA genomic stability differs between Li23 and HuH-7 cells. Furthermore, we observed for the first time that GC content in nonstructural regions increased for codon optimization in human cells. Moreover, we demonstrated that the long-term cultured genome-length HCV RNA-replicating cells were useful as a library source for the isolation or characterization of resistant HCVs against anti-HCV agents.

Using Li23-derived cell culture systems, we observed that the mutation rates of HCV RNAs were 4.0–9.0×10^−3^ and 2.7–4.0×10^−3^ base substitutions/site/year in 5′-terminus-NS2 regions and NS3-NS5B regions, respectively. These values were 2.1–6.4 times and 1.4–2.9 times higher than those (1.4–1.9×10^−3^ base substitutions/site/year) previously obtained in chimpanzees [15], [16] and in a patient [14] with chronic hepatitis C. Since we previously found that the mutation rates of genome-length HCV RNAs were 4.4–7.4×10^−3^ and 2.5–3.7×10^−3^ base/substitutions/site/year in 5′-terminus-NS2 regions and NS3-NS5B regions, respectively, using HuH-7-derived cell culture systems [21], most of the mutation rates were proved not to change, regardless of the cell type. Since the selective pressures of the humoral immune responses [17] targeting the envelope proteins and cellular immune responses [18] targeting all HCV proteins function *in vivo*, the mutation rates obtained using the cell culture systems without the immunological pressure would be reasonable values as a potential mutation rate of HCV in RNA replication.

Thus far, many studies using the HCV replicon system, including the whole-virus system of JFH-1 strain HCV, have clarified the aa positions that are essential for the efficient HCV reproduction [32]–[34]. On the basis of those reports, we made lists of functional aas in HCV genotype 1 (partly genotype 2a) (Tables S1 and S2) and then checked whether the position of each functional aa was the same as the position of the aa substitution detected in this study. This investigation revealed that most of the functional aas were conserved during the 4-year culture of genome-length HCV RNA-replicating cells, suggesting that the basic HCV RNA replication mechanism does not change during long-term cell culture. However, as we observed several aa substitutions in the Core from OL11 series, the function of the Core may be lost in long-term-cultured OL11 cells, although the Core is not essential for RNA replication.

Although our report is the only one to conduct genetic variation and diversity analyses of HCV during the long-term HCV RNA replication of genotype 1b in cell culture, several similar reports use long-term HCV RNA (JFH-1 strain of genotype 2a)-replicating HuH-7-derived cells [35]–[41]. In those studies, many adaptive mutations were found as the result of long-term persistent HCV reproduction. Although it is a bit complicated to decide the corresponding aa positions exactly, as the O strain and JFH-1 strain belong to different genotypes, we examined whether the substituted aas detected in this study were found in those adaptive mutations obtained from reports using the JFH-1 strain. We noticed that only I414T substituted between 2- and 4-year cultures of OL cells was the same aa substitution as the JFH-1 strain (Table S3). It is unlikely that this substitution functions as an adaptive mutation for RNA replication because the HCV RNA level decreased between 2- and 4-year cultures (Fig. 1 and [26]). It is also unlikely that this substitution increases virus production because virus particles were not produced from the cells cultured for 2 or 4 years (Fig. S1). However, we can exclude the possibility that other aa substitutions detected at the corresponding positions to the JFH-1 strain are adaptive mutations.

In our previous study using HuH-7-derived cell culture systems, we noticed that none of the aa substitutions were detected in the N-terminal half (242 aa of aa 1976 to 2217) of the NS5A after 2-year cultures, suggesting that this region would be the most critical for maintaining RNA replication. However, we detected many aa substitutions in this region in all Li23-derived cell lines after 2-year or 4-year cultures (Table 3). These were the following aa substitutions: L2003F and H2057R in OL series; R1978K, D1979E, K1998R, S2079Y, and K2212R in OL8 series; K2050R, F2099Y, and T2217I in OL11 series; L2125V in OL14 series. These results suggest that the N-terminal half of NS5A also possesses further variability to allow a better environment for HCV RNA reproduction. Another interesting feature we noticed is that several aa substitutions were spontaneously detected in the interferon (IFN) sensitivity determining region (ISDR) [42] (aa 2209–2248) and in the IFN/Ribavirin (RBV) resistance-determining region (IRRDR) [43] (aa 2334–2379) of NS5A in the cells without IFN or RBV treatment. In ISDR, K2212R (OL8 series), T2217I (OL11 series), D2220G (OL8 and OL14 series), and S2246P (OL series) were detected. Furthermore, in IRRDR, T2336S (OL11 series), S2338P (OL series), S2342P (OL8 series), L2347R (OL8 series), T2351A (OL11 series), F2352V (OL8 series), F2352S (OL11 series), F2352L (OL14 series), S2355P (OL series), S2355T (OL14 series), T2364A (OL8 series), P2369H (OL series), S2373P (OL14 series), D2374N (OL14 series), and D2377G (OL8 series) were detected (Table 3). These aa substitutions except for D2220G also appeared in a seemingly random manner, although aa 2352 and 2355 were hot spots for aa substitutions in the Li23-derived cell culture system but not in the HuH-7-derived cell culture system [20]. These results suggest that the sensitivity to IFN or RBV might change during long-term cell culture, although it has not yet been proved that variations in ISDR or IRRDR may change the sensitivity to IFN or RBV.

When we explored this possibility, we newly noticed that L2003F (L31F in NS5A) was detected as a conservative aa in OL(2Y) and OL(4Y) cells. F in aa 2003 has been reported as an aa showing low-level resistance to daclatasvir (BMS-790052), an NS5A inhibitor that will soon serve as a clinical cure [44]. Furthermore, V1081A (V55A in NS3) was also detected as a conservative aa in OL(4Y) cells. A in aa 1081 has been reported as an aa showing low-level resistance to boceprevir, an NS3-4A serine protease inhibitor that was approved as a new direct-acting antiviral drug [45]. These facts indicate that clones resistant to anti-HCV agents emerge naturally without treatment. Since V1081A and L2003F were detected in all HCV clones derived from OL(4Y) cells, these aa substitutions may possess some advantage for cell proliferation. Furthermore, as a minor population, a larger number of resistant HCV clones may emerge from such a long-term cell culture. Although neither daclatasvir nor boceprevir was available in this study, we demonstrated that telaprevir-treated OL(4Y) cells completely and easily converted a telaprevir-sensitive phenotype into a telaprevir-resistant phenotype without a decrease in the level of HCV RNA replication, suggesting that telaprevir-resistant HCV clones rapidly became dominant populations in the telaprevir-treated OL(4Y) cells.

As well as V1081A and L2003F, we noticed for the first time that D2292E (D320E in NS5A) appeared in OL(2Y), OL(4Y), OL14(2Y), and OL14(4Y) cells as a conservative aa substitution, although our previous study using HuH-7-derived cells detected D2292E as a conservative aa substitution after 2-year cultures of genome-length HCV RNA-replicating OB and OE cells [20]. It has been reported that D2292E is an aa substitution that causes resistance to cyclosporine (CsA) and other cyclophilin inhibitors, including NIM811 and DEB025 [46], [47]. These facts also indicate that the HCV species possessing D2292E substitution can become the main species naturally in cultured cells without CsA or other treatments.

This study demonstrated that a single genome-length HCV RNA could exhibit a remarkable diversity after 4 years in cell culture with RNA replication. Our results, together with previous results, suggest that such diversity of HCV obtained by long-term cell culture may be useful not only for understanding the genetic variations and diversity of HCV but also for the examination of the resistant spectrum of anti-HCV agents.

## Supporting Information

Figure S1 **No infectious virus production from long-term cultured genome-length HCV RNA-replicating cells.** HCV infection to RSc (1×10^4^) and ORL8c (5×10^3^) cells was performed using the supernatant (each 1 ml after filtering through a 0.20-μm filter [Kurabo, Osaka, Japan]) of OL(0Y), OL(4Y), OL8(4Y), OL11(4Y), or OL14(4Y) cells as an inoculum, as described previously [23]. As a positive control, HCV JFH-1 virus was used for the infection at a multiplicity of infection of 0.1 or 1.0. At 7 days and 8 days, (A) the levels of Core in the supernatant after filtering through a 0.20-μm filter were quantified by enzyme-linked immunosorbent assay (Mitsubishi Kagaku Bio-Clinical Laboratories, Tokyo, Japan) and (B) the levels of intracellular HCV RNA were quantified by LightCycler PCR, as described previously [21], [27].
(TIF)

Figure S2 **Phylogenetic trees of deduced aa in ORF of genome-length HCV RNA populations obtained in long-term cell culture.** The phylogenetic trees are depicted on the basis of aa sequences deduced from all cDNA clones obtained by 0-year, 2-year, and 4-year cultures of OL, OL8, OL11, and OL14 cells. (A) The Core-NS2 regions in ORF of genome-length HCV RNA. O/C-2 indicates the original aa sequences of the Core-NS2 regions in ORF of ON/C-5B/QR,KE,SR RNA [21]. (B) The NS3-NS5B regions in ORF of genome-length HCV RNA. O/3-5B/QR,KE,SR indicates the original aa sequences of the NS3-NS5B regions in ORF of ON/C-5B/QR,KE,SR RNA [21].
(TIF)

Table S1 **Comparative list of functional aas in HCV genotype 1 and aa substitutions detected in this study (I).**
(DOC)

Table S2 **Comparative list of functional aas in HCV genotype 1 and aa substitutions detected in this study (II).**
(DOC)

Table S3 **Hereditary aa substitutions detected in persistent HCV JFH-1 (genotype 2a) infection; comparison with aa substitutions detected in this study.**
(DOC)
