# Supplementary figures and images for: Genetic Characterization of Hepatitis C Virus in Long-Term RNA Replication Using Li23 Cell Culture Systems
Source: PLoS One. 2014 Mar 13;9(3):e91156. doi: 10.1371/journal.pone.0091156 (PMC3953375; doi:10.1371/journal.pone.0091156)

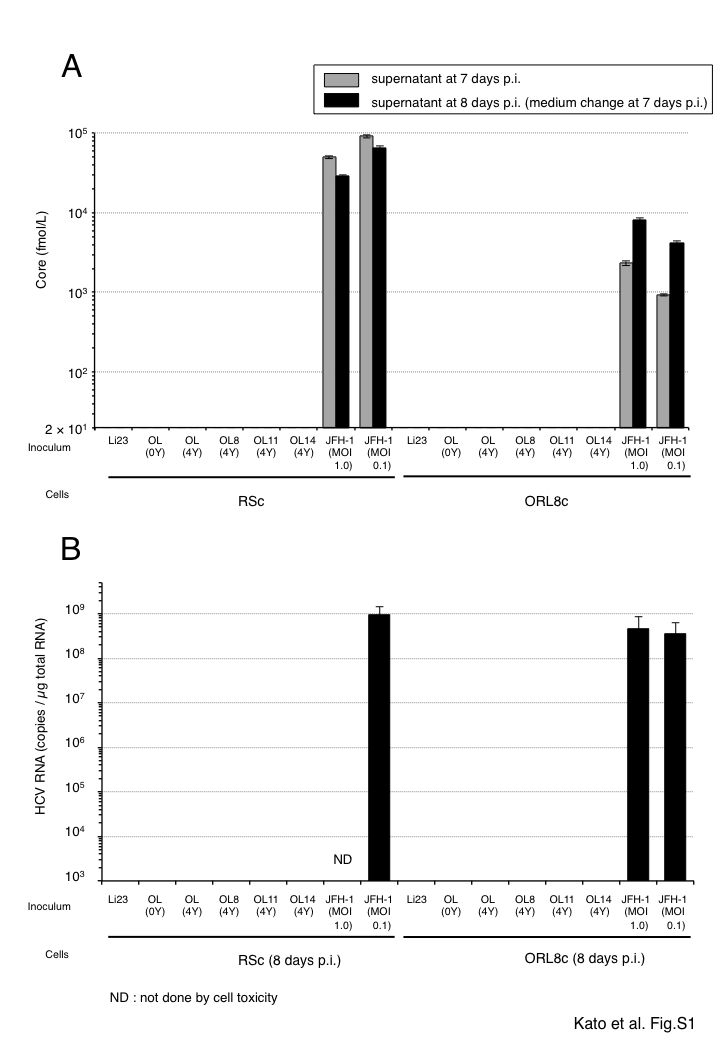

Supplement: Figure S1 — No infectious virus production from long-term cultured genome-length HCV RNA-replicating cells. HCV infection to RSc (1×10^4^) and ORL8c (5×10^3^) cells was performed using the supernatant (each 1 ml after filtering through a 0.20-μm filter [Kurabo, Osaka, Japan]) of OL(0Y), OL(4Y), OL8(4Y), OL11(4Y), or OL14(4Y) cells as an inoculum, as described previously [23]. As a positive control, HCV JFH-1 virus was used for the infection at a multiplicity of infection of 0.1 or 1.0. At 7 days and 8 days, (A) the levels of Core in the supernatant after filtering through a 0.20-μm filter were quantified by enzyme-linked immunosorbent assay (Mitsubishi Kagaku Bio-Clinical Laboratories, Tokyo, Japan) and (B) the levels of intracellular HCV RNA were quantified by LightCycler PCR, as described previously [21], [27]. (TIF) [file pone.0091156.s001.tif]

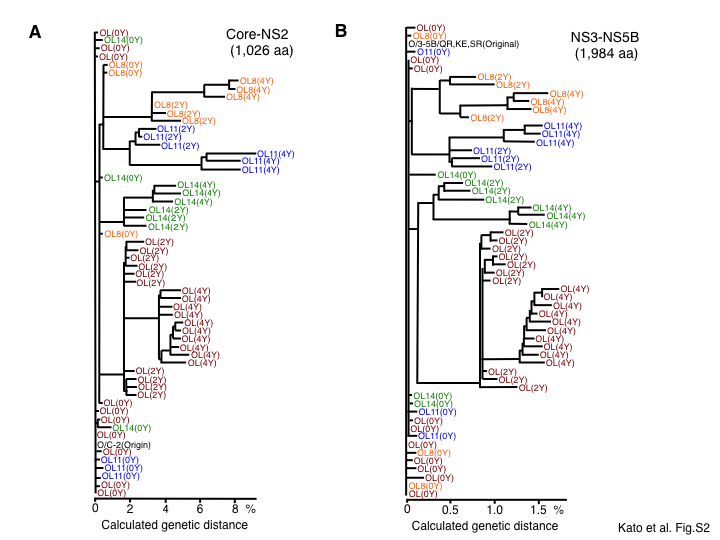

Supplement: Figure S2 — Phylogenetic trees of deduced aa in ORF of genome-length HCV RNA populations obtained in long-term cell culture. The phylogenetic trees are depicted on the basis of aa sequences deduced from all cDNA clones obtained by 0-year, 2-year, and 4-year cultures of OL, OL8, OL11, and OL14 cells. (A) The Core-NS2 regions in ORF of genome-length HCV RNA. O/C-2 indicates the original aa sequences of the Core-NS2 regions in ORF of ON/C-5B/QR,KE,SR RNA [21]. (B) The NS3-NS5B regions in ORF of genome-length HCV RNA. O/3-5B/QR,KE,SR indicates the original aa sequences of the NS3-NS5B regions in ORF of ON/C-5B/QR,KE,SR RNA [21]. (TIF) [file pone.0091156.s002.tif]
