# Supplementary material for: Genetic Characterization of Hepatitis C Virus in Long-Term RNA Replication Using Li23 Cell Culture Systems
Source: PLoS One. 2014 Mar 13;9(3):e91156. doi: 10.1371/journal.pone.0091156 (PMC3953375; doi:10.1371/journal.pone.0091156)
Supplement: Table S1 — Comparative list of functional aas in HCV genotype 1 and aa substitutions detected in this study (I). (DOC) [file pone.0091156.s003.doc]

Supporting Information Table

Table S1. Comparative list of functional aas in HCV genotype 1 and aa substitutions detected in this study (I)

| Position of functional aa(Region) | Original | OL | OL8 | OL11 | OL14 | Function |
| --- | --- | --- | --- | --- | --- | --- |
| 139(Core) | L |  |  | **P** |  | Cleavage by signal peptide peptidase |
| 140(Core) | V |  |  |  |  | Cleavage by signal peptide peptidase |
| 144(Core) | L |  |  |  |  | Cleavage by signal peptide peptidase |
| 125-144(Core) | TLTCGFADLMGYIPLVGAPL | L(133) |  | S(125) |  | Targeting Core to lipid droplet |
|  |  |  |  | **S**(133) |  |  |
|  |  |  |  | T(134) |  |  |
|  |  |  |  | P(139) |  |  |
| 149-158(Core) | RALAHGVRVL |  |  | T(150) |  | Targeting Core to mitochondria |
| 195/197(E1) | N/S |  |  |  |  | Glycosylation |
| 209/211(E1) | N/S |  |  |  |  | Glycosylation |
| 234/236(E1) | N/S |  |  |  |  | Glycosylation |
| 250/252(E1) | N/S |  |  |  |  | Glycosylation |
| 305/307(E1) | N/S |  |  |  |  | Glycosylation |
| 370(E1) | K |  |  |  |  | Retention to endoplasmic reticulum |
| 417/419(E2) | N/S |  | **D**(417) |  | **R**(419) | Glycosylation |
| 420(E2)^b^ | W |  |  |  |  | Binding to CD81 |
| 423/425(E2) | N/T |  |  |  |  | Glycosylation |
| 448/450(E2) | N/S | P(450) |  |  |  | Glycosylation |
| 488(E2)^a^ | H |  |  |  |  | Binding to heparin sulfate  SR-BI-dependent entry |
| 523(E2)^b^ | G | **S** |  |  |  | Binding to CD81 |
| 526(E2)^b^ | T |  |  |  |  | Binding to CD81 |
| 527(E2)^b^ | Y |  |  |  |  | Binding to CD81 |
| 529(E2)^b^ | W |  |  |  |  | Binding to CD81 |
| 530(E2)^b^ | G |  |  |  |  | Binding to CD81 |
| 532/534(E2) | N/T | G(532) |  |  |  | Glycosylation |
| 535(E2)^b^ | D |  |  |  |  | Binding to CD81 |
| 540/542(E2) | N/T |  |  |  |  | Glycosylation |
| 556/558(E2) | N/T | **S**(556) |  |  |  | Glycosylation |
| 576/578(E2) | N/T |  |  |  |  | Glycosylation |
| 623/625(E2) | N/T |  |  | S(623) |  | Glycosylation |
| 645/647(E2) | N/T |  |  |  |  | Glycosylation |
| 648(E2)^a^ | R |  |  |  |  | Binding to heparin sulfate  SR-BI-dependent entry |
| 728/730(E2) | D/R |  |  |  |  | Retention to endoplasmic reticulum |
| 952/972/993(NS2) | H/E/C |  |  |  |  | NS2/3 processing |

Conservative aa substitutions detected after 2-year and 4-year cultures are shown in boldface.

The blank shows that the original aa has not changed.

All data except a and b were obtained from collection of review articles [32].

^a^ Data from the study [33] using HCV JFH-1 (genotype 2a).

^b^Data from the recent study [34].
