# Supplementary material for: Genetic Characterization of Hepatitis C Virus in Long-Term RNA Replication Using Li23 Cell Culture Systems
Source: PLoS One. 2014 Mar 13;9(3):e91156. doi: 10.1371/journal.pone.0091156 (PMC3953375; doi:10.1371/journal.pone.0091156)
Supplement: Table S2 — Comparative list of functional aas in HCV genotype 1 and aa substitutions detected in this study (II). (DOC) [file pone.0091156.s004.doc]

Supporting Information Table

Table S2. Comparative list of functional aas in HCV genotype 1 and aa substitutions detected in this study (II)

| Position of functional aa(Region) | Original | OL | OL8 | OL11 | OL14 | Function |
| --- | --- | --- | --- | --- | --- | --- |
| 1083/1107/1165(NS3) | H/D/S |  |  |  |  | Catalytic triad of NS3 protease |
| 1123/1125/1171/1175(NS3) | C/C/C/H |  |  |  |  | Zn-binding residues |
| 1161/1180/1183(NS3) | L/F/A |  |  |  |  | S1 pocket residues of NS3 protease |
| 1149/1187/1191(NS3) | R/R/K |  |  |  |  | S6 pocket residues of NS3 protease |
| 1419(NS3) | R |  |  |  |  | Unwinding of RNA |
| 1464/1470(NS3) | F/F |  |  |  |  | Release of DNA by ATP binding |
| 1519/1527(NS3) | E/W |  |  |  |  | Importance to helicase activity |
| 1680/1682/1686/1688(NS4A) | V/I/I/L |  |  |  |  | Interaction with NS3 |
| 1840-1846(NS4B) | GSIGLGK |  |  |  |  | Nucleotide binding |
| 1922/1932(NS4B) | F/T |  |  |  |  | Nucleotide binding |
| 1939-1942(NS4B) | DAAA |  |  |  |  | Nucleotide binding |
| 1977-2002(NS5A) | SGSWLRDVWDWICTVLTDFKTWLQSKLLPR |  | **K**/E/R(1978/1979/1998) |  |  | Membrane anchor |
| 2011/2029/2031/2052(NS5A) | C/C/C/C |  |  |  |  | Zn-binding residues |
| 2135-2139(NS5A) | PACKP |  |  |  |  | Interaction with NS4A |
| 2194/2197/2201/2204(NS5A) | S/S/S/S |  |  |  |  | Hyperphosphorylation |
| 2210-2248(NS5A) | SLKATCTTHHDSPDADLIEANLLWRQEMGGNITRVESEN | P(2246) | R/**G**(2212/2220) | **I**(2217) | **G**(2220) | Interferon sensitivity Determining region |
| 2334-2379(NS5A) | ILTESTVSSALAELATKTFGSSGSSAVDSGTATAPPDGPSDDGDAG | P/**P**/H(2338/2355/2369) | P/R/V/A/G(2342/2347/2352/2364/2377) | **S**/A/**S**(2336/2351/2352) | L/T/P/N(2352/2355/2373/2374) | Interferon/ribavirin Resistance-determining region |
| 2577/2786/2805/2809/2813(NS5B) | R/S/R/T/R |  |  |  |  | Catalytic NTP binding pocket |
| 2736-2738(NS5B) | GDD |  |  |  |  | Active site |

Conservative aa substitutions detected after 2-year and 4-year cultures are shown in boldface.

The blank shows that the original aa has not changed.

All data except a and b were obtained from collection of review articles [32].
