# Supplementary material for: Genetic Characterization of Hepatitis C Virus in Long-Term RNA Replication Using Li23 Cell Culture Systems
Source: PLoS One. 2014 Mar 13;9(3):e91156. doi: 10.1371/journal.pone.0091156 (PMC3953375; doi:10.1371/journal.pone.0091156)
Supplement: Table S3 — Hereditary aa substitutions detected in persistent HCV JFH-1 (genotype 2a) infection; comparison with aa substitutions detected in this study. (DOC) [file pone.0091156.s005.doc]

Supporting Information Table

Table S3. Hereditary aa substitutions detected in persistent HCV JFH-1 (genotype 2a) infection; comparison with aa substitutions detected in this study

| Aa substitution (Region) | Period of cell culture | Functional Characteristics | Aa in  genotype 1 (position) | OL | OL8 | OL11 | OL14 | Ref. no |
| --- | --- | --- | --- | --- | --- | --- | --- | --- |
| K74T(Core) | 119 days | NC | R(74) |  |  |  |  | [35] |
| G451R(E2) | “ | Adaptive mutation | G(451) |  |  |  |  | “ |
| M1051T(NS3) | “ | NC | L(1047) |  |  |  |  | “ |
| C2219R(NS5A) | “ | “ | C(2215) |  |  |  |  | “ |
| K74Q(Core) | 160 days | “ | R(74) |  |  |  |  | “ |
| V388P(E2) | “ | “ | S(388) |  |  |  |  | “ |
| I414T(E2) | “ | “ | I(414) | T |  |  | **Δ** | “ |
| L644I(E2) | “ | “ | L(640) |  |  |  |  | “ |
| N16D(Core) | cp8+sp8 | “ | N(16) |  |  |  |  | [36] |
| I374T(E1) | cp3 | “ | V(374) |  |  |  |  | “ |
| V2440L(NS5A) | cp3 | Adaptive mutation | V(2417) |  |  |  |  | “ |
| L2468S(NS5B) | 5 months | “ | L(2445) |  |  |  |  | [37] |
| K78E(Core) | cp38,47 | Adaptive mutation^a^ | Q(78) |  |  |  |  | [38] |
| T396A(E2) | cp27,38,47 | “^a^ | T(396) |  |  |  |  | “ |
| T416A(E2) | “ | “^a^ | T(416) |  |  |  |  | “ |
| N534H(E2) | “ | “^a^ | N(532) | G |  |  |  | “ |
| A712V(E2) | “ | “^a^ | V^b^(708) |  |  |  |  | “ |
| Y852H(NS2) | “ | “^a^ | Y(848) |  |  |  | **Δ** | “ |
| W879R(NS2) | “ | “^a^ | L(875) |  |  |  |  | “ |
| F2281L(NS5A) | “ | “^a^ | K(2277) |  |  | R |  | “ |
| M2876L(NS5A) | “ | “^a^ | L^b^(2853) | I |  |  |  | “ |
| T2925A(NS5B) | cp47 | “^a^ | N(2902) |  |  |  |  | “ |
| K74T(|Core) | Numerous passage | Adaptive mutation | R(74) |  |  |  |  | [39] |
| P251L(E1) | “ | “ | S(251) |  |  | G | G | “ |
| Q1012R(NS2) | “ | “ | R^b^(1008) |  |  |  |  | “ |
| I1162V(NS3) | “ | “ | V^b^(1158) |  |  |  |  | “ |
| T1209A(NS3) | “ | “ | M(1205) |  |  | V |  | “ |
| F2004I(NS5A) | “ | “ | L(2000) |  |  |  |  | “ |
| V2089L(NS5A) | “ | “ | V(2085) |  |  |  |  | “ |
| V2250A(NS5A) | “ | “ | S(2246) | P |  |  |  | “ |
| P2271L(NS5A) | “ | “ | E(2267) |  |  |  |  | “ |
| K2320E(NS5A) | “ | “ | K(2320) | R |  | R |  | “ |
| K2350T(NS5A) | “ | “ | K(2350) |  |  |  |  | “ |
| D2360A(NS5A) | “ | “ | V(2360) |  |  |  |  | “ |
| V2440L(NS5A) | “ | “ | V(2417) |  |  |  |  | “ |
| F2004I(NS5A) | 120 days | “ | L(2000) |  |  |  |  | [40] |
| I2270T(NS5A) | “ | NC | R(2266) |  |  |  |  | “ |
| C2274T(NS5A) | “ | Adaptive mutation | V(2270) |  | A |  |  | “ |
| Y2293N(NS5A) | “ | NC | N/A | N/A | N/A | N/A | N/A | “ |
| E2337K(NS5A) | “ | Adaptive mutation | E(2337) |  |  |  |  | “ |
| I2349T(NS5A) | “ | NC | T^b^(2349) |  |  |  |  | “ |
| G2362V(NS5A) | “ | “ | S(2362) |  |  |  |  | “ |
| Q2412K(NS5A) | “ | “ | P(2389) |  |  |  |  | “ |
| D2437N(NS5A) | “ | Adaptive mutation | E(2414) |  |  |  |  | “ |
| V2440L(NS5A) | “ | “ | V(2417) |  |  |  |  | “ |
| H2505Q(NS5B) | 130 days | “ (for O strain) | H(2482) |  |  |  |  | [41] |
| V2995L(NS5B) | “ | Adaptive mutation | A(2972) |  |  |  |  | “ |

NC: not clarified; cp: culture passage; sp: supernatant passage; N/A: not applicable.

^a^ A set of all mutations in [36] was important for the enhancement of HCV replication.

^b^ Same as the aa substituted in the HCV-JFH-1 strain.

Conservative aa substitutions detected after 2-year and 4-year cultures are shown in boldface.

The blank shows that the original aa has not changed.
